# The potential of hydrogel‐free tumoroids in head and neck squamous cell carcinoma

**DOI:** 10.1002/cam4.70129

**Published:** 2024-08-22

**Authors:** Michael Wong, Sarju Vasani, Omar Breik, Xi Zhang, Lizbeth Kenny, Chamindie Punyadeera

**Affiliations:** ^1^ Saliva & Liquid Biopsy Translational Laboratory Griffith Institute for Drug Discovery, Griffith University Nathan Queensland Australia; ^2^ Department of Otolaryngology, Head and Neck Surgery Royal Brisbane and Women's Hospital Herston Queensland Australia; ^3^ School of Clinical Medicine The University of Queensland Brisbane Queensland Australia; ^4^ Department of Oral and Maxillofacial Surgery Royal Brisbane and Women's Hospital Herston Queensland Australia; ^5^ Cancer Care Services Royal Brisbane and Women's Hospital Herston Queensland Australia; ^6^ Menzies Health Institute Queensland, School of Medical Science Griffith University Gold Coast Campus Southport Queensland Australia; ^7^ Translational Research Institute Woolloongabba Queensland Australia

**Keywords:** head and neck oncology, hydrogels, squamous cell carcinoma, tumour organoids

## Abstract

**Background:**

Head and neck malignancy, and in particular squamous cell carcinoma (SCC), is responsible for a significant disease burden globally. The lack of an optimal in vitro model system to accurately recapitulate in vivo response to therapy in HNSCC remains a challenge. The development of patient‐derived three‐dimensional tumour cultures, or tumoroids, has enabled improved modelling of the tumour microenvironment through simulation of important characteristics such as tumour hypoxia, cell–cell interactions and nutrient diffusion characteristics.

**Methods:**

We performed a comprehensive English‐language literature review of current methods of tumoroid development utilising Matrigel and Cultrex Basement Membrane Extract 2 (key terms: tumour organoids, tumoroids, hydrogels, Matrigel, Cultrex, squamous cell carcinoma, head and neck)—two common proprietary murine‐derived hydrogels containing extracellular matrix proteins. Nascent literature on the establishment of a novel hydrogel‐free platform for tumoroid development as distinct from these existing methods was also explored.

**Results:**

Whilst useful for facilitating cell‐matrix interactions and providing a scaffold for three‐dimensional cell growth and organisation, murine‐derived cell matrix methods were noted to have notable limitations including temperature sensitivity and the medium forming a barrier to analysis of the supernatant. A novel hydrogel‐free method of establishing in vitro tumoroid cultures has been subject to experimentation in colorectal but not head and neck malignancy. The absence of a hydrogel provides for the de novo synthesis of extracellular matrix native to the tumour and self‐organisation of cells within this scaffold through the use of ultralow attachment plates. This model demonstrates similar structural and physiological properties to native tissue, whilst enabling more accurate biomimicry of the tumour microenvironment for drug testing.

**Conclusions:**

In the absence of prior experimentation on a hydrogel‐free method for establishing HNSCC tumoroids, and comparisons between hydrogel and hydrogel‐free models, the future development of a comparative protocol encompassing recruitment, collection, processing and analysis represents a valuable opportunity.

## 1 INTRODUCTION

Head and neck malignancy contributes to significant morbidity and mortality globally and is the seventh most common type of malignancy by incidence.^1^, ^2^ According to the latest GLOBOCAN estimates, it has a global incidence of more than 890,000 individuals annually and is attributable to in excess of 450,000 deaths.^3^ It has three times the incidence in males compared to females and two‐thirds of reported head and neck cancers occur in developing nations.^1^ This number has experienced significant growth across all geographical regions over the past 40 years, particularly in males and for malignancies of the oropharynx.^4^ In parts of South‐East Asia, and Central and Eastern Europe particularly, this figure may be as high as 20 per 100,000 population.^5^

Per capita in Australia, head and neck malignancy commands a higher incidence as the sixth most common type of malignancy, with 5189 new diagnoses and 1247 deaths in Australia in 2022.^6^, ^7^ In keeping with local population growth, the overall incidence of head and neck malignancy in Australia has grown from 2472 cases in 1982 to 4884 cases in 2018,^6^ however, age‐standardised incidence rates have remained largely stable. Deaths have also increased over this time in spite of a decrease in the age‐standardised mortality rate from 6.1 per 100,000 population in 1982 to 3.6 per 100,000 population in 2020.^6^

Squamous cell carcinoma (SCC) is the most common type of head and neck malignancy comprising more than 90% of cases, with tobacco use and human papillomavirus (HPV) infection implicated as strong risk factors.^8^ This makes head and neck SCC (HNSCC) an important target for research given its prevalence and significant disease burden.^9^ Although various treatment modalities are used in the treatment of HNSCC—including surgical excision, radiotherapy, and chemotherapy—the challenges and failures of treatment still leave significant scope for improved treatments and personalised medicine.^9^ The lack of an optimal in vitro model system to accurately determine response to treatment in HNSCC remains a challenge.

The ability of three‐dimensional (3D) cell cultures to mimic in vivo physiology including cell–cell and cell‐matrix interactions—and their responses to chemotherapeutic agents—have broad implications in drug discovery and development.^10^ Tumour organoids, or tumoroids, are patient‐derived primary 3D cell cultures that can be developed into lineages of genetically stable cells and kept stable over long time periods.^10^ Recent advances have seen the development of tumoroids arising from the gastrointestinal tract, brain, lung, breast and head and neck.^9^, ^10^

The acquisition of data on treatment response unique to a patient's cancer may be realised through the characterisation of reliable and reproducible methods of tumoroid culture.^9^ This could be used in future to guide the development of personalised treatment regimens for head and neck cancers based on the in‐vitro response of a patient's tumour when tested against various chemotherapeutic agents.^9^ This review article describes the current and novel methods in HNSCC tumour organoid development and the implications these may have on the future development of new protocols and their relevance to precision medicine.

## 2 EPIDEMIOLOGY

HNSCC of the upper aerodigestive tract can arise from the mucosal epithelium of the sinonasal cavity, nasopharynx, oral cavity, oropharynx, larynx and hypopharynx.^1^, ^11^ Since 1980 there has been both a decrease in the incidence of laryngeal SCC and an increase in SCC of the oral cavity and oropharynx, particularly in the under 40 age group.^1^, ^12^, ^13^, ^14^ This has been attributed to the rise of HPV infection in this population secondary to changes in sexual practises and a trend towards more sexual partners per capita in developed nations,^1^ resulting in a higher rate of HPV‐related SCCs.

Classically, SCC of the upper aerodigestive tract was stereotypically seen in males aged >60 years with a history of long‐term tobacco and alcohol use.^12^ In a departure from this paradigm, those affected by HPV‐positive SCC tend to be in a younger male cohort in which smoking remains a major, although less prominent, risk factor. This group also tends to be of higher socioeconomic status in developed nations with these cancers having identifiable clinical and pathological differences to HPV‐negative SCC.^12^, ^13^ These include clinical characteristics such as early tumour staging and advanced nodal staging, and pathological idiosyncrasies including basaloid morphology and poorer differentiation.^1^, ^11^, ^12^ High‐risk HPV subtypes HPV‐16, 18, 31 and 33 are most common causative viral subtypes, with HPV‐16 responsible for more than 90 percent of malignancies.^5^, ^12^, ^15^ Although the prevalence of HPV in oropharyngeal SCC (OPSCC) differs geographically, there has been an overall rise in HPV‐related OPSCC to approximately 40% globally and up to 73% in the United States since the 1990s.^5^ The median age at diagnosis continues to rise in accordance with the birth cohort effect.^4^

Other major risk factors for SCC of the upper aerodigestive tract include tobacco use, alcohol consumption, and betel nut chewing.^16^ Tobacco products are most commonly consumed in the form of cigarettes; however, other forms of tobacco smoking and chewing are also well‐established carcinogenic factors.^5^ This increases the risk of HNSCC up to 25 times, with higher risk linked to greater exposure.^5^ Alcohol consumption also increases cancer risk and its combination with tobacco use is synergistic.^1^ The rise of betel nut chewing, particularly in South‐East Asian countries, is also implicated with increased risk of up to 15 times.^5^ Less common risk factors for HNSCC include poor oral hygiene, chronic Hepatitis C and HIV infection, and other forms of immunosuppression.^5^

## 3 ONCOGENESIS AND MOLECULAR BIOLOGY

The inactivation of tumour suppressor genes, including CDKN2A encoding for p16 and TP53 encoding for p53, is a key pathway in HNSCC oncogenesis.^11^ The use of p16 expression as a surrogate marker for HPV infection in the characterisation of HNSCC is now well established.^16^, ^17^ p16 is a protein responsible for arresting cell cycle progression through the inhibition of retinoblastoma pocket protein (pRb) phosphorylation via the cyclin D‐CDK4/6 complex.^18^ This enables pRb to sequester E2F, thereby preventing its translocation to cell nuclei to promote transcription of proteins required for cell‐cycle progression.^18^ In HPV‐negative HNSCC, reduced expression of p16 and p53 enable uninhibited cell proliferation.^11^, ^18^ This can occur subsequent to the mutation or inactivation of CDKN2A from the deletion of chromosomal region 9p21, in addition to the mutation or inactivation of TP53. Paradoxically, in HPV‐related disease, there is overexpression of p16 without a concomitant arrest of cell cycle progression secondary to HPV‐induced degradation of pRb.^18^ HPV viral oncoproteins E6 and E7 are responsible for this effect of bypassing physiological mechanisms of tumour suppression.^1^

Oncogenes and growth factor receptors are other molecular factors involved in tumorigenesis. Cyclin D1 is an oncogene amplified in up to half of head and neck malignancies, and is associated with reduced time to disease recurrence, more advanced staging and reduced survival.^1^ Epidermal growth factor receptor (EGFR), a transmembrane protein responsible for regulating cell growth via the RAS–RAF‐mitogen activated protein kinase (MAPK) pathway, has also been found to be overexpressed in HNSCC and associated with nodal metastasis.^19^

## 4 DIAGNOSIS, TREATMENT AND PROGNOSIS

Mucosal HNSCC has a plethora of clinical presentations according to site, often at varying stages of disease progression. Initial presentation may include upper aerodigestive tract symptoms such as dysphonia, dysphagia, odynophagia and otalgia, in addition to constitutional symptoms and altered sensation.^11^, ^13^ Airway obstruction, difficulty eating and the obvious sensation or visualisation of a mass can also be present.^11^

The diagnosis of SCC must be confirmed through tissue biopsy, ideally of the primary tumour if identified and accessible, but alternatively using fine needle aspiration (FNA) or core biopsy of a regional neck lymph node.^11^ HPV status pertaining to malignancy of the oropharynx, or in the case of the unknown primary, may be determined through p16 immunohistochemistry (IHC), the detection of HPV DNA through polymerase chain reaction (PCR) or in‐situ hybridisation (ISH), or the detection of viral oncoprotein E6 and E7 mRNA.^11^

The main primary treatment modalities for HNSCC are surgical excision, chemotherapy, and radiotherapy. Immunotherapy is used in metastatic disease. Single modalities or combinations thereof may be utilised. Treatment deintensification in p16‐positive disease to maintain outcomes whilst reducing treatment‐related morbidity is under active investigation with multiple trials in progress.^13^ These include the study of outcomes with single modalities and the de‐escalation of adjuvant therapy.^13^, ^20^ Immunotherapy for head and neck SCC is also a nascent development in treatment and continues to be under active investigation. The PD‐1/PD‐L1 checkpoint is a promising target as upregulation of PD‐L1 as seen particularly in advanced HNSCC reduces T‐cell mediated apoptosis.^11^ As of the first half of 2023, only anti PD‐1/PD‐L1, and CTLA4 antibodies are in routine clinical use.^13^

## 5 THE NEED FOR IN VITRO PATIENT‐DERIVED TUMOUR MODELS

**FIGURE 1:**
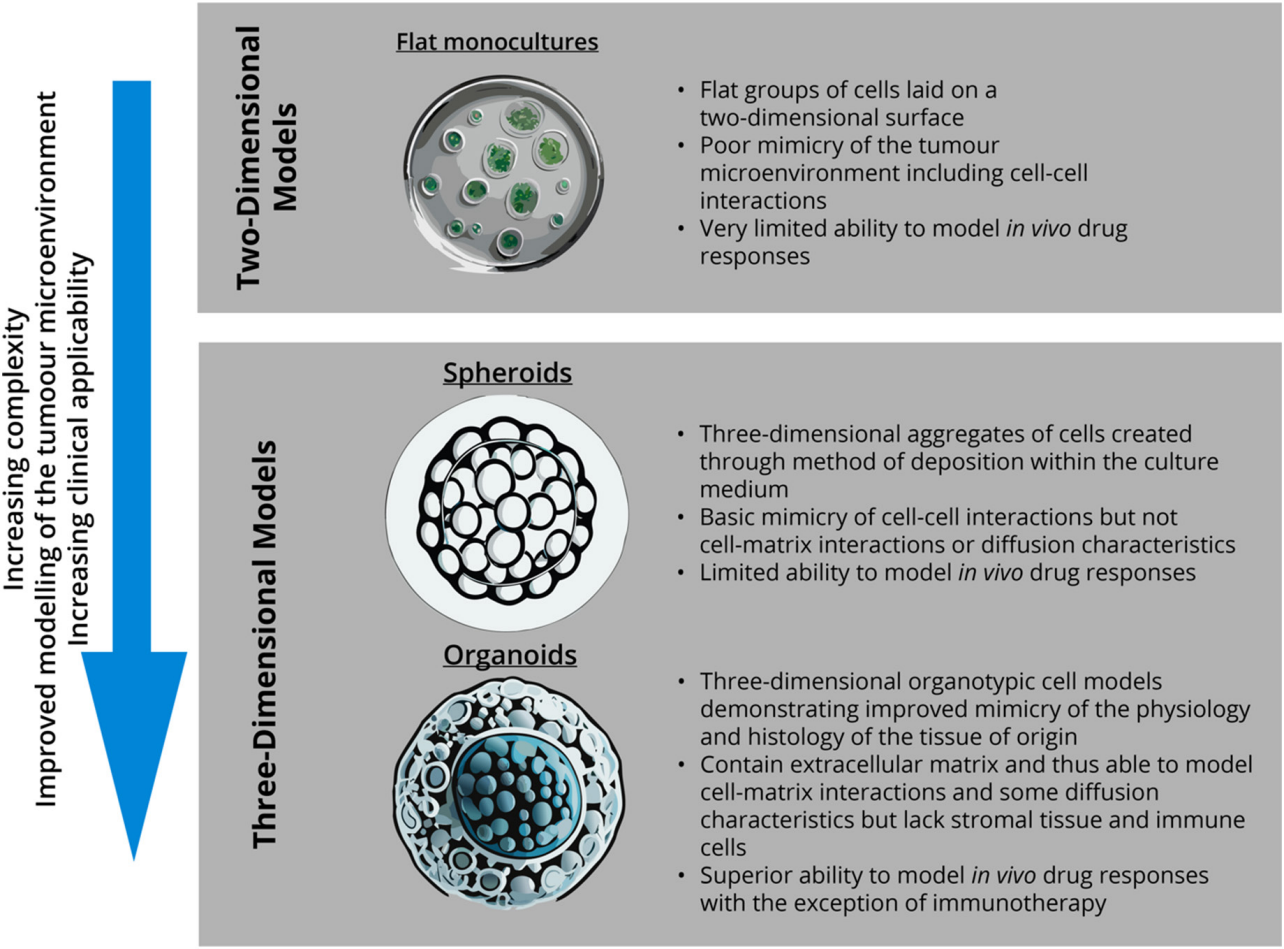
Comparison of the main categories of tissue‐derived in vitro cell culture models (Figure created by first author with referenced information).^23^, ^24^, ^25^

The tumour microenvironment (TME)—especially the interactions between tumour cells, immune cells and fibroblasts—is gaining traction as a means to explain drug resistance in cancer.^21^ Two‐dimensional (2D) cell cultures, whilst relatively straightforward and inexpensive to culture in vitro,^22^ demonstrate limited ability to mimic the TME and are therefore less predictive of cancer treatment responses in vivo (Figure 1).^26^, ^27^ Lee et al. recently demonstrated for HNSCC that the utilisation of extracellular matrix (ECM) in 2D cell sheets may provide for increased longevity.^22^, ^23^

Spheroids are simple 3D cell cultures that result in spherical aggregates of cells typically of a single cell type.^28^ This aggregation is achieved artificially, and spheroids only resemble a part of their tissue of origin, have limited capacity to reproduce or be developed into lineages, and poorly mimic the behaviour of native tumour.^23^, ^27^, ^28^ Lacking an ECM and the morphology of tumour, they generally do not simulate the tumour microenvironment well, particularly cell‐matrix interactions and molecular diffusion characteristics.^27^, ^28^ The utilisation of HNSCC‐spheroids to model drug testing is a relatively new endeavour, with most methods using HNSCC cell lines (Table 1). Whilst dose‐dependent drug responses can be observed in spheroids, generally these responses do not simulate those in vivo, are not informative or generalisable, or can be inconsistent with the results expected (Table 1). A paper by Tanaka et al.^32^ is one of few examples in the literature in which spheroid‐derived cells from HNSCC have been successfully used to predict chemotherapeutic drug responses in vivo to cisplatin and docetaxel. However, spheroids are poorly predictive in vitro models for drug testing and development.^24^

**TABLE 1:** Overview of 3D spheroid models of mucosal HNSCC used in anti‐cancer therapy testing.

Authors	Cell type(s)	Tumour Site(s) of origin	Spheroid model media	Study aim	Outcome
Kadletz et al. (2015)^29^	HNSCC cell lines FaDu, CAL27 and SCC25	FaDu—hypopharynx, CAL27—tongue, SCC25—tongue	DMEM/F12, EGF, bFGF	Compare immunohistochemical differences between 2D monolayer cell cultures and spheroids	Spheroids developed from all three cell lines. FaDu had greatest spheroid size, shape and intercellular adherence. Higher IC_50_ in spheroids compared to 2D cultures. Response to radiotherapy in 2D cultures only and not spheroids. Variable expression of EGFR, VEGFR and Ki67 in spheroids compared to 2D cultures.
Braunholz et al. (2016)^30^	HNSCC cell lines FaDu, SCC‐9, UT‐SCC‐9P, and UT‐SCC‐9R	FaDu—hypopharynx, SCC‐9—tongue, UT‐SCC‐9P, UT‐SCC‐9R—CTX resistant subclone of 9P	Anti‐adhesive polymer poly‐HEMA	Determine role of EGFR in formation and anchorage‐independent survival of spheroids	FaDu had highest spheroid‐forming potential (6 spheroids/well) with SCC‐9 having the lowest potential (1 spheroid/well). Spheroid‐formation found to be EGFR dependent whilst extent of formation found to be cell‐line dependent. Cetuximab and gefitinib inhibited anchorage‐independent cell survival and spheroid formation.
Hagemann et al. (2018)^31^	Proprietary HNSCC cell line PiCa	PiCa—proprietary cell line (site not provided)	DMEM, BEGM, 10% FBS 1% penicillin/streptomycin, 1% sodium pyruvate, 1% non‐essential amino acids, 1% L‐glutamine	Standardised establishment of spheroid cultures for chemoradiotherapy testing in vitro from proprietary cell line	Reproducible spheroid generation from single cell suspension with greater reliability using an ultra‐low attachment plate method over a hanging drop method. Significant decrease in spheroid size with cisplatin and 5‐fluorouracil. No synergistic effect with addition of radiotherapy on spheroid size although a decrease in viable cell count was seen.
Tanaka et al. (2018)^32^	Patient‐derived HNSCC	Buccal mucosa, floor of mouth, gingiva, tongue, tonsil, pharynx, larynx, metastatic lymph nodes	Matrigel®, StemPro hESC supplemented with bFGF	Development of cancer tissue‐originated spheroids (CTOS) with methodology successfully established in colorectal, lung and bladder cancer for drug testing	CTOS success rate of 30.2% with 53.8% success of establishing 2D cell lines from CTOS. Similar IC_50_ for cisplatin and docetaxel in vivo (mice) compared to when calculated in vitro.
Ayuso et al. (2019)^33^	HNSCC UM‐SCC‐1 (HPV negative) and UM‐SCC‐47 (HPV positive) cell lines, cancer‐associated fibroblasts (CAF)	UM‐SCC‐1—floor of mouth, UM‐SCC‐47—lateral tongue	3D collagen hydrogel comprising: PBS, NaOH, collagen type I, cell suspension	Comparison of 2D (± CAF co‐culture) and 3D in vitro HNSCC models for drug testing with cetuximab and AZD8055	HPV‐cells had greater drug resistance (cetuximab and AZD8055) in CAF co‐culture and 3D spheroids. HPV+ cells responded more consistently across methods.
Melissaridou et al. (2019)^34^	HNSCC cell lines LK0858B, LK0902, LK0917, LK1108 and LK1122	LK0858B—tongue, LK0902—tongue, LK0917—gingiva, LK1108—hypopharynx, LK1122—larynx	Keratinocyte‐SFM supplemented with antibiotics (penicillin 50 U/mL, streptomycin 50 μg/mL), and 10% FBS	Assess effect of 2D and 3D cell cultures on treatment response, and epithelial‐mesenchymal transition (EMT) and cancer stem cell (CSC) genes	Variable spheroid size and density according to cell line. Upregulation of CDH1, NANOG and SOX2 in spheroids compared to 2D cultures. Spheroids more resistant to cisplatin and cetuximab
Azharuddin et al. (2019)^35^	HNSCC cell lines LK0912, LK0917, and LK1108	LK0902—tongue, LK0917—gingiva, LK1108—hypopharynx	Keratinocyte‐SFM supplemented with antibiotics (penicillin 50 U/mL, streptomycin 50 μg/mL), and 10% FBS	Utilisation of spheroids to monitor drug uptake and cytotoxicity in the detection of multi drug resistance compared to 2D cultures	Higher IC_50_ values with spheroids compared to 2D cultures. Doxorubin demonstrated least drug sensitivity with both 2D and spheroid culture methods compared to cisplatin and methotrexate. Drug efflux pump activity assay possible for all three cell lines
Magan et al. (2020)^36^	HNSCC cell lines LK0902, LK0917 and LK1108, CAF	LK0902—tongue, LK0917—gingiva, LK1108—hypopharynx	Keratinocyte‐SFM supplemented with antibiotics (penicillin 50 U/mL, streptomycin 50 μg/mL) and 10% FBS	Develop a biologically relevant HNSCC‐CAF co‐culture in vitro model to predict treatment response and investigate the effect of CAF	Greater spheroid cell growth with CAF, increased EGFR expression. Greater response to cetiximab in spheroids with greater EGFR expression. Paradoxically increased cell proliferation marker Ki67 and EGFR expression in spheroids post cisplatin exposure.
Tenschert et al. (2022)^37^	HNSCC cell lines UMSCC‐11A, UMSCC‐11B, UMSCC‐22B, and UD‐SCC‐01, CAF	UMSCC‐11A—larynx, UMSCC‐11B—larynx, UMSCC‐22B—hypopharynx, UD‐SCC‐01—oropharynx	EMEM, 10% FCS, penicillin, streptomycin, amphotericin B. DMEM for CAF culture	Determine optimal conditions to culture and utilise spheroids derived from HNSCC cell lines, optimal timing of use, effects of co‐culture with CAF, and responses to cisplatin	3 out of 4 cell lines formed spheroids. UD‐SCC‐01 cells did not. CAF did not increase viable cell number. Round‐bottomed wells supported single spheroid formation. Fewer remaining viable cells post cisplatin exposure at day 2 after spheroid formation (6%) compared to day 8 (24%)

In recent years, tumour organoids have demonstrated recapitulation of the in vivo genetic and phenotypic characteristics of cancers arising from various human tissue sites in the respiratory tract, gastrointestinal tract, and reproductive system.^38^, ^39^ In particular, organoids are superior at resembling the tumour microenvironment—including in vivo cell–cell interactions, oxygen transfer, and nutrient and waste diffusion and transport.^25^, ^40^ The presence of ECM in these 3D structures further contributes to architectural similarity with tissues in vivo, and therefore better mimicry of tumour cell interactions with other cell types present. This is especially relevant as cell types other than tumour cells can be added to organoid cultures, co‐existing within the ECM, and interactions with these other cell types also contribute to the TME.^23^ Organoids also demonstrate better replication of tissue responses when exposed to chemotherapeutic agents, given that tumour cell interactions with normal host cells influence their susceptibility, when they are co‐present in patient‐derived tissue and the resulting cell culture.^25^, ^26^, ^39^

The use of organoids in head and neck cancer is a relatively new endeavour, with prior research predominantly focussed on the development of 2D cell lines.^38^ There are few examples in the literature of the development of 3D cell lines in head and neck cancer, with variable success demonstrated to date.^25^, ^38^ Organoids are more costly, and complex to grow and maintain but have been shown to have superior applicability to precision medicine in head and neck SCC in their nascent phase.^28^

## 6 DEVELOPMENT OF HNSCC TUMOROID MODELS

Driehuis et al. in 2019 were one of the first groups to establish a successful end‐to‐end protocol for the development and characterisation of patient‐derived oral mucosal tumoroids derived‐from HNSCC and subsequent in vitro testing with chemoradiotherapy.^38^ Their methodology utilised two commercially available hydrogels or synthetic extracellular matrices—Cultrex™ BME 2 and Matrigel®—in conjunction with a growth factor medium to develop 31 successful tumoroid lines.^38^ In brief, the protocol involves digestion of the patient‐derived tumour tissue using trypsin to yield a cell slurry. The cells were then processed and grown in Advanced DMEM with added GlutaMAX, penicillin‐steptomycin and HEPES, and seeded in organoid medium containing the hydrogels and supplemented with growth factors including EGF, FGF‐2, FGF‐10, PGE2, R‐spondin and Noggin. Media changes were performed every 2–3 days with organoid splitting after 1–2 weeks. Growth rate analysis occurred at day 7 through quantitative cell count.^38^

This study, in conjunction with subsequent studies, have been able to demonstrate a number of key characteristics important to HNSCC tumoroid development and testing. Tumoroid lines were established and maintained long‐term comprising over 15 passages, cryopreservation and recovery.^38^ They have been found to demonstrate functional, histopathological and molecular characteristics specific to HNSCC.^38^ This included varying tumour morphology with growth under microscopy as seen in the original tumours, immunohistochemical similarity, genetic instability and alterations,^41^ and the ability to infect cells with HPV in vitro. TP53 and CDKN2A mutations were able to be productively assessed in vitro and tumoroids were found to have a heterogeneous cellular composition mimicking in vivo tumours.^38^

HNSCC tumoroids were also subjected to in vitro testing with radiation and chemotherapeutic drugs including cisplatin, carboplatin and cetixumab.^38^ Although average IC_50_ values for cisplatin (5.91 μmol/L) and carboplatin (44.1 μmol/L) were different, correlation between their sensitivities was seen.^38^ Responses to EGFR‐inhibitor cetixumab were variable with no statistically significant correlation demonstrated between EGFR expression and cetuximab.^38^ However, responses to radiation in vitro were found to be predictive of in vivo patient radioresistance with curative intent radiotherapy. Three patients with post‐radiotherapy recurrence also had tumours with the greatest extent of in vitro radioresistance.^38^ Importantly, the synergism of chemoradiotherapy was demonstrated.^38^ Successful tumoroid xenotransplantation subcutaneously into mice was also performed, yielding macroscopically visible tumours after 6 weeks. The mice were sacrificed, with tumours then fixed in formaldehyde, qualitatively demonstrating histological characteristics of HNSCC including nuclear atypia and mitoses.^38^

Subsequently, a number of studies have been able to demonstrate the reliability of HNSCC tumoroids as a predictor for treatment response in vivo (Table 2).^25^, ^28^, ^44^, ^46^, ^47^ In particular, tumoroids have been shown to be capable of mimicking tumour hypoxia, which is negatively predictive of response to chemotherapy and radiotherapy.^25^, ^48^, ^49^ 3D tumour models better mimic tumour hypoxia over 2D models as like in vivo, not all cells are contained at the surface and exposed to atmospheric levels of oxygen.^50^, ^51^

**TABLE 2:** Overview of organoid models of mucosal HNSCC used in oncologic therapy testing.

Authors	Cell type(s)	Tumour site(s) of origin	Organotypic model media	Study aim	Outcome
Zhao et al. (2017)^42^	Patient‐derived tongue SCC and cancer‐associated fibroblasts (CAF), CAL27 cell line	Tongue, CAL27—human tongue SCC	Matrigel®, DMEM/F12, FBS, animal‐derived (mice, pigs, rats) tongue extracellular matrix (TEM)	Development of a 3D tongue SCC model for drug testing using scaffold (animal TEM)	Qualitative histological demonstration of organised invasion of TEM over 28 days. No effect of TEM on tongue SCC cells on cisplatin chemosensitivity
Tam et al. (2018)^43^	PCI‐13 HNSCC cell line and human natural killer (NK) cells	PACI‐13—Oral cavity	Human NK cell and PCI‐13 co‐culture using Cultrex™ Rat Collagen I, F12, Solution C, DMEM media containing 10% FBS and IFN‐γ +/− doxycycline	Determine whether tumour‐derived CEACAM1 (a cell‐membrane receptor expressed in malignant cells) inhibits NK cell cytotoxicity and whether blockade of CEACAM1 restores antitumor immunity	CEACAM1‐positive HNSCC cells had increased size and number of organoids and were more resistant to NK cell‐mediated cytotoxicity. Cell viability was 89.8% ascertained via DAPI staining. Inhibition of CEACAM1 with shRNA restored cytoxicity.
Driehuis et al. (2019)^38^	Patient‐derived HNSCC	Oral cavity (floor of mouth, tongue, and gingiva/alveolar process), pharynx, larynx, salivary gland, nasal cavity, and neck	BME, Advanced DMEM +/+/+, GlutaMAX, HEPES, Penicillin, Streptomycin, human EGF, human FGF10, Prostaglandin E2, CHIR, R‐spondin, Noggin	Development of organotypic HNSCC mucosal tumour organoids for drug testing	31 successful tumoroid lines with ~60% success rate. Tumoroids demonstrated IHC similarity to tumours of origin with similar genetic mutations. In vitro responses seen with cisplatin and carboplatin, but not cetuximab
Kijima et al. (2019)^44^, ^45^	Patient‐derived oesophageal and oropharyngeal SCC and adjacent normal mucosa	Oesophagus, oropharynx	DMEM/F12, GlutaMAX, HEPES, Gentamicin, Penicillin, Streptomycin, recombinant human EGF, Noggin/R‐spondin	Development of patient‐derived oesophageal and oropharyngeal SCC and normal tissue organoids to assess feasibility, drug testing with 5‐fluorouracil	15 successful organoid lines (71.4% success rate) matching the histopathology of the tumours of origin. Patients whose tumours developed successful organoids tended to be more chemoradioresistant. 5‐fluorouracil resistance associated with high CD44 expression.

Tumours in vivo adapt to hypoxic conditions associated with rapid growth by modulating hypoxia‐inducible factors (HIF) and vascular endothelial growth factor (VEGF).^49^, ^52^ This increases expression of EGFR, conferring treatment resistance and cell longevity, as exemplified in a study on HNSCC cell lines by Boeckx et al.,^49^ in which increased cetuximab sensitivity was noted in a cell line exposed to hypoxia for 72 h. IC_50_ values for cetuximab with normoxia, 24 and 72 h of hypoxia were 2.38 ± 0.59 nM, 0.64 ± 0.38 nM, and 0.10 ± 0.05 nM respectively.^49^

To date, tumoroid models have used a number of tissues from patient‐derived HNSCC and salivary gland epithelium, and patient derived cancer‐associated fibroblasts.^25^, ^53^ There are however few examples of research into the development of tumoroids from patient‐derived mucosal HNSCC specifically, and all methods of culturing HNSCC tumoroids to this point have only involved the use of synthetic ECM—most commonly Matrigel® and Cultrex™.^25^ Whilst the TME also comprises the interactions of tumour cells with immune cells and endothelial cells, and is more than simply the presence of ECM or the type used, the inclusion of matrix is key to the biochemical, physicochemical and mechanical properties of the environment in which tumour cells reside.^54^ This relates to PDTO development in that the tumour cells themselves and cells present in co‐cultures like CAF render these properties architecturally and histologically more closely to the in vivo state.^23^ Different cell types cannot optimally interact without the existence of such a scaffold and the resultant three‐dimensional cell organisation giving rise to greater physiological relevance.^54^

## 7 LIMITATIONS OF CURRENT TUMOROID MODELS

Current methods utilising hydrogels introduce challenges in the sampling and analysis of cells, including the measurement of growth factors within medium.^55^ Solidification of the medium, whilst necessary for providing the intended effect of ECM scaffolding, limits access to secreted growth factors and messengers that may be desired for analysis.^55^ Further to this, the medium can serve as a barrier to introduced chemotherapeutic agents used for sensitivity testing.

A further limitation of tumoroids in their relevance to precision medicine occurs through their absence of stromal tissue and immune cells.^48^ This especially limits their utility in testing cancer immunotherapy in‐vitro.^48^ The presence of immune cells is a further contributor to functional modelling of inflammatory responses to oncogenesis and recapitulating in vivo treatment responses.^56^ Natural killer (NK) cells have a role in immunological surveillance and their presence in patient‐derived tumoroid co‐cultures has been demonstrated to decrease radioresistance in HNSCC.^57^ Cancer‐associated fibroblasts (CAF) have been linked to interleukin‐6 (IL‐6) and VEGF expression and subsequent tumour angiogenesis in tumoroid co‐cultures.^58^ Functional inactivation of leucocytes including CD4 and CD8 T‐cells, mast cells, dendritic cells and macrophages has been associated with altered expression of inflammatory factors including ITGA5, OLR1, CCL5 and CXCL8, resulting in a poorer prognosis.^56^

The ORGAVADS trial proposed in March 2023 is the most recent proposal in the literature for the development of tumoroids from HNSCC and again utilises Matrigel® and Cultrex™ BME 2 as hydrogels without a hydrogel‐free arm.^59^ The trial aims to further assess in vitro responses of patient‐derived tumoroids to radiotherapy, chemotherapy and importantly, immunotherapy.^59^ The proposed methodology aims to incorporate co‐culture of tumoroids with autologous clonally expanded tumour‐specific T‐cells.^59^ These are identified by the reactivity and activation of markers CD137, CD107a and IFN‐γ through flow cytometry. Tumoroid responses to immunotherapeutic agents nivolumab and pembrolizumab added to the co‐culture are evaluated qualitatively through LAMP‐1 CD8+ T‐cell membrane protein visualisation and Incucyte S3 timelapse, and quantitatively through caspase‐3 cleavage and the CellTiter‐Glo 3D cell viability assay.^59^ In vitro treatment responses are then compared to the in vivo responses of the patients.^59^

Most methods describing the use of hydrogels in the development of human tissue‐derived organoids utilise Matrigel®.^60^ Whilst these protocols have been well described and established for its use, Matrigel® is a relatively undefined medium with more than 1800 proteins.^60^ Therefore, the role of these proteins in the development and function of organoid lines is poorly understood and a likely contributor to the variation seen in the literature in the success of Matrigel®‐based cultures.^60^ It is not known, or currently easily discernible, what elements of its composition may contribute to cell‐signalling and the maintenance of the tumour microenvironment. Further to this, as Matrigel® and Cultrex™ are derived from murine sarcomas, their utility in the culture of human cells is limited and it is likely they lack vital components for successful organoid culture and immunogenicity.^60^ From an ethical standpoint, and especially if these methods are to be eventually employed at scale, we should also not discount the issues of sustainability and welfare from the use of animal‐derived products in cell culture.^61^

## 8 IDEAL CHARACTERISTICS OF TUMOROID MEDIA

The ideal medium for tumoroid culture is one in which the tumour microenvironment is most accurately represented. Native ECM is complex and so too are cell–cell interactions in tissues—particularly whole organs or those with multiple cell types.^54^, ^60^ Three‐dimensional structure is also key to modelling the in vivo effects of tumour hypoxia and diffusion characteristics, which confers metastatic ability and resistance to chemoradiotherapy.^29^, ^49^, ^50^ A number of key attributes are considered imperative to the efficacy of tumoroid media as described by Gan et al.^54^ Cell adhesion peptides contribute to cell organisation and facilitate inter‐cellular signalling and subsequent differentiation.^54^, ^62^ Good ECM substitutes also mimic the viscoelastic properties of native tissue, which includes the response of the tissue to applied mechanical force and tension.^54^, ^63^, ^64^, ^65^ These are the stressors that cells and ECM are subject to in vivo with ambulation and the hydrostatic pressure of blood flow. Ideally, the substitute ECM should be responsive to tissue expansion and growth.^64^

Organogenesis is also reliant on chemical messengers comprising growth factors, cytokines and chemoattractants involved in the recruitment of immune cells which then have a role in gene expression.^66^, ^67^ These include morphogens WNT, BMP and NOG, in which their gradients lead to spatial organisation and polarisation of cells.^66^, ^68^ Gene expression in turn supports cell metabolic function resulting in mutation, growth, and proliferation. Temperature and pH stability of the media in accordance with physiological parameters is also necessary for protein stability and maintenance of function.^63^ This occurs through the creation of hydrogen and electrostatic bonds, and the effects of hydrophobic phenomena.^63^ Altogether, the key mechanical and biochemical properties of ECM are seen as vital in the success of any tumoroid media, however current methods are limited by their poor biomimicry of these characteristics.^67^

## 9 HYDROGEL‐FREE TUMOROID MODELS

The murine‐derived nature of existing hydrogel‐based tumoroid models contributes to their limitations in simulating the human TME and recapitulate in vivo treatment responses. Hydrogel‐free methods of 3D cell culture utilise media that lacks tissue‐derived, synthetic or substitute ECM.^69^ These methods were first trialled in spheroid development and involve the use of various cell seeding techniques in the absence of ECM, including spinner flasks, magnetic levitation, hanging drops and ultralow attachment plates.^69^ They function by enabling self‐organisation of individual cells into aggregates through the promotion of intercellular interactions and inhibition of cell interactions with substrate.^69^ Hydrogel‐free methods are relatively simple and straightforward to set‐up, and scale with good long‐term culture viability.^69^ Ultralow attachment plates have been further demonstrated to provide high output. However, these methods are also associated with significant heterogeneity in the size and shape of the organotypic cultures.^69^

The use of these techniques in tumour organoids is novel, having only yet been employed for cancer types such as colorectal cancer,^70^ and not yet in HNSCC. The colorectal cancer models comprised the development of hydrogel‐free tumoroids utilising ultralow attachment plates.^27^, ^70^ Although this method is insufficiently explored in the literature to draw a conclusion, early results have revealed that tumoroids developed without hydrogel are morphologically and functionally similar to those developed with hydrogel, and can likewise be utilised for in vitro drug testing.^70^

An additional benefit of the hydrogel‐free model is that it is particularly useful for the addition, collection and analysis from supernatant of secreted growth factors and extracellular vesicles.^27^, ^70^ Drugs can also be easily added to cultures for testing. By allowing cells to self‐organise and synthesise their own ECM, there is in theory no better artificial or derived ECM substitute, enabling superior biomimicry of the tumour microenvironment. It should be noted however that many of the limitations of current hydrogel‐based methods, such as the absence of immune cells and mechanical stressors, remain in hydrogel‐free methods.^69^ To date, there is no evidence in the literature for hydrogel free models in HNSCC, or a comparison between hydrogel and hydrogel‐free models.

## 10 CONCLUSION–THE FUTURE ROLE OF HYDROGEL‐FREE TUMOROIDS IN PRECISION MEDICINE

The full benefit of head and neck tumoroids in drug development is yet to be realised. Utilising patient‐derived tissue comes with obvious benefits over animal models in their relevance to precision medicine. Current hydrogel‐based methods of tumoroid culture have already shown great promise in their ability to replicate the tumour microenvironment and as a predictive platform for drug and radiotherapy experimentation. As the process of drug‐development begins with in vitro testing, tumour organoids represent an opportunity for characterisation of drug sensitivity, resistance, dose, cytotoxicity and molecular targets in this important first step.^25^

In the absence of prior literature specific to hydrogel‐free HNSCC tumoroids, and comparisons between hydrogel and hydrogel‐free models, the future development of a comparative protocol encompassing recruitment, collection, processing and analysis represents a valuable opportunity. The potential for reproducibility this could afford is necessary in further methodological validation and future utilisation of these models in precision medicine. Whilst the novel hydrogel‐free model does not eliminate the disadvantages of existing organoid models, it could potentially serve as an iterative improvement to our understanding of 3D cell cultures not utilised before in HNSCC.

## PRECIS

Existing methods of three‐dimensional tumour organoid culture when applied to head and neck squamous cell carcinoma utilise murine‐derived hydrogels which with several limitations in their ability to recapitulate the tumour microenvironment. A novel hydrogel‐free method may hold promise in precision medicine.

## AUTHOR CONTRIBUTIONS

**Michael Wong:** Conceptualization (lead); data curation (lead); investigation (lead); project administration (lead); resources (lead); software (lead); validation (lead); visualization (lead); writing – original draft (lead); writing – review and editing (lead). **Sarju Vasani:** Conceptualization (lead); data curation (equal); funding acquisition (lead); investigation (equal); methodology (equal); project administration (equal); resources (lead); supervision (lead); validation (equal); visualization (equal); writing – original draft (equal); writing – review and editing (equal). **Omar Breik:** Conceptualization (supporting); investigation (equal); project administration (equal); resources (equal); supervision (equal); writing – review and editing (supporting). **Xi Zhang:** Conceptualization (equal); funding acquisition (equal); investigation (equal); project administration (equal); resources (equal); supervision (equal); validation (equal); visualization (equal); writing – review and editing (equal). **Lizbeth Kenny:** Conceptualization (supporting); project administration (supporting); resources (supporting); supervision (supporting); validation (supporting); visualization (supporting); writing – review and editing (supporting). **Chamindie Punyadeera:** Conceptualization (lead); funding acquisition (lead); investigation (equal); project administration (lead); resources (lead); supervision (lead); validation (equal); visualization (equal); writing – review and editing (equal).

## FUNDING INFORMATION

Chamindie Punyadeera is currently funded by the National Institutes of Health (R21EB030349), Cancer Australia (APP1145657), the National Health and Medical Research Council (APP2002576 and APP2012560), the Collaborative Research Grants‐Metro North Health, and the Garnett Passe and Rodney Williams Foundation.

## CONFLICT OF INTEREST STATEMENT

The authors have no conflicts of interest to declare.

## DISCLOSURE

Please note that the pictorial elements of Figure 1 were in part created in Adobe Illustrator using generative artificial intelligence (AI) features within the paid software. These are permitted for commercial use. None of the text within the figures, tables, or entirety of this article has been created in whole or part with generative AI.

## Data Availability

Data sharing is not applicable to this article as no new data were created or analyzed in this study.
